# Randomized double-blind placebo-controlled trial of sublingual immunotherapy in children with house dust mite allergy in primary care: study design and recruitment

**DOI:** 10.1186/1471-2296-9-59

**Published:** 2008-10-20

**Authors:** Cindy MA de Bot, Heleen Moed, Marjolein Y Berger, Esther Röder, Hans de Groot, Johan C de Jongste, Roy Gerth van Wijk, Johannes C van der Wouden

**Affiliations:** 1 Department of General Practice, Erasmus MC-University Medical Center Rotterdam, PO Box 2040, 3000 CA Rotterdam, the Netherlands; 2 Department of Allergology, Erasmus MC-University Medical Center Rotterdam, PO Box 2040, 3000 CA Rotterdam, the Netherlands; 3 Department of Pediatric Respiratory Medicine, Erasmus MC-University Medical Center/Sophia Children's Hospital, Rotterdam, PO Box 2040, 3000 CA Rotterdam, the Netherlands

## Abstract

**Background:**

For respiratory allergic disorders in children, sublingual immunotherapy has been developed as an alternative to subcutaneous immunotherapy. Sublingual immunotherapy is more convenient, has a good safety profile and might be an attractive option for use in primary care. A randomized double-blind placebo-controlled study was designed to establish the efficacy of sublingual immunotherapy with house dust mite allergen compared to placebo treatment in 6 to18-year-old children with allergic rhinitis and a proven house dust mite allergy in primary care. Described here are the methodology, recruitment phases, and main characteristics of the recruited children.

**Methods:**

Recruitment took place in September to December of 2005 and 2006. General practitioners (in south-west Netherlands) selected children who had ever been diagnosed with allergic rhinitis. Children and parents could respond to a postal invitation. Children who responded positively were screened by telephone using a nasal symptom score. After this screening, an inclusion visit took place during which a blood sample was taken for the RAST test.

**Results:**

A total of 226 general practitioners invited almost 6000 children: of these, 51% was male and 40% <12 years of age. The target sample size was 256 children; 251 patients were finally included. The most frequent reasons given for not participating were: absence or mildness of symptoms, absence of house dust mite allergy, and being allergic to grass pollen or tree pollen only. Asthma symptoms were reported by 37% of the children. Of the enrolled children, 71% was sensitized to both house dust mite and grass pollen. Roughly similar proportions of children were diagnosed as being sensitized to one, two, three or four common inhalant allergens.

**Conclusion:**

Our study was designed in accordance with recent recommendations for research on establishing the efficacy of sublingual immunotherapy; 98% of the target sample size was achieved. This study is expected to provide useful information on sublingual immunotherapy with house dust mite allergen in primary care. The results on efficacy and safety are expected to be available by 2010.

**Trial registration:**

the trial is registered as ISRCTN91141483 (Dutch Trial Register)

## Background

Specific immunotherapy with allergens might prevent the onset of asthma in individuals with allergic rhinitis and may accelerate the remission of asthma in children with allergic disease. [1-3] Although subcutaneous immunotherapy (SCIT) is an effective treatment of respiratory allergic disorders, [4] the injections can be uncomfortable and side effects, though rare, may be serious and even fatal. [5,6] The use of specific sublingual immunotherapy (SLIT) for treatment of respiratory allergic disorders in children may be a viable alternative to SCIT because of its convenient form of administration and good safety profile – which has allowed home administration of SLIT. [7,8] Thus, although SLIT seems particularly suitable for children in primary care, most clinical trials up to now have been performed in a hospital setting.

Evidence for the efficacy of SLIT in children remains inconclusive. Various reviews concluded that there was insufficient evidence to recommend SLIT for use in routine clinical practice. [9-11] In their Cochrane review, Wilson et al. concluded that SLIT is an accepted treatment for adults; studies with children revealed no significant reduction in symptoms and medication scores, but the number of participants was small. [12]

In 2001, the Allergic Rhinitis and its Impact on Asthma (ARIA) guidelines were published in co-operation with the World Health Organization. [13] They recommend treatment of allergic rhinitis in a stepwise manner (using a combination of allergen avoidance, pharmacotherapy and immunotherapy) based on the duration and severity of disease, rather than on the type of exposure (i.e. seasonal, perennial, occupational) as in previous guidelines. [14] Immunotherapy is recommended for patients with more severe disease, for those not responding to usual treatments, or for those refusing usual treatments; this type of patient is generally treated in a hospital setting and/or by a specialist.

In the Netherlands, allergic rhinitis in children is usually managed by the general practitioner (GP). We hypothesized that SLIT could be an effective treatment in primary care and designed a study to evaluate the efficacy and safety of SLIT in children and adolescents with house dust mite-induced allergic rhinitis. Here we describe the methodology, recruitment, and main characteristics of the primary care study population.

## Methods

### Study design

This ongoing study is a randomized double-blind placebo-controlled study, comparing the efficacy of SLIT with house dust mite allergen (SLIT-HDM) to that of placebo treatment in 6 to 18-year-old children with allergic rhinitis and a proven house dust mite allergy in primary care. Patients entered the study and started treatment either in September-December 2005 or in September-December 2006 for a period of approximately two years. Written informed consent was obtained. The study was approved by the Ethical Review Board of Erasmus MC-University Medical Center Rotterdam. The trial was registered as ISRCTN91141483.

### Participants and recruitment

GPs in south-western Netherlands selected children aged 6 to 18 years in their computerized patient files with either a diagnosis of hay fever/allergic rhinitis or relevant medication use: i.e. antihistamines for systemic use; nasal corticosteroids; topical decongestants; topical anti-allergics, and other nasal preparations.

Recruitment took place September to December in 2005 and in 2006. An information letter signed by the GP was sent to the selected children. This letter described the general purpose of the study, elicited cooperation, and provided a return form and envelope. On the return form children and parents could indicate whether or not they were interested in the study; if not interested they could indicate the reason for not participating.

Participants who responded positively were telephoned by a research assistant to arrange a screening interview (see below). The research assistant asked questions about nasal symptoms during the last three months, the history of allergic rhinitis, general medication use, and use of asthma medication. Table 1 gives an overview of all inclusion and exclusion criteria.

**Table 1:** Inclusion and exclusion criteria for the study population

**Inclusion criteria**
• aged 6–18 years
• history of allergic rhinitis for at least 1 year
• IgE antibodies ≥0.7 kU/l to house dust mite
• no use of nasal steroids in the month before start of baseline measurements
• rhinitis symptom score of at least 4 out of 12 during last 3 months
• signed informed consent
**Exclusion criteria**
• severe asthma (requiring 800 mcg budesonide daily or equivalent for other inhaled steroids; or requiring >3 courses of oral prednisone/prednisolone in previous year or required hospital stay for asthma in previous year)
• sensitization to pets present at home (IgE antibodies ≥0.7 kU/l)
• planned surgery of nasal cavity
• having received immunotherapy in past 3 years
• language barrier
• contraindications to sublingual immunotherapy (as supplied by the manufacturer)

After telephone screening an inclusion visit took place for those who met the inclusion/exclusion criteria and who agreed (children/parents) to further participation. During this visit, the research assistant performed/recorded the following: rhinitis symptoms during the last month and last week (nasal symptoms: rhinorrhea, blocked nose, sneezing, itching); conjunctivitis symptoms during the last month and last week (eye symptoms: tearing, itching, redness); International Study of Asthma and Allergies in Childhood (ISAAC) questionnaire [15] for rhinitis and asthma; wheeze and cough; family history of allergy, asthma and eczema; rhinoconjunctivitis-specific quality of life for pediatrics and adolescents (PRQLQ and AdolRQLQ[16,17]); blood sample for RAST (grass pollen, tree pollen, HDM, cat dander and a pet, if present at home) (CAP-Phadiatop^®^, Pharmacia Diagnostics AB, Uppsala, Sweden); and physical examination (weight and height).

After the screening visit, when children met the inclusion criteria and none of the exclusion criteria and children and parents agreed to participate, a home visit was scheduled to provide instructions about the baseline diary. Every day for one month, children recorded the symptoms related to allergic rhinitis on a diary card; also reported were other complaints, rescue medication, and other medication needed (see below). At this visit the research assistant took dust samples from the child's bedroom floor and mattress to assess indoor HDM exposure. This will be repeated after two years.

After the baseline diaries had been completed a new visit was scheduled and, after signing informed consent, participants were assigned to SLIT treatment or placebo according to the randomization schedule (see below).

### Randomization

Randomization was generated by a computer program in varying block sizes unknown to the investigators. The randomization list was passed to the Department of Pharmacy at Erasmus MC. In order to ensure that disease severity was similar between patients assigned to verum therapy and those assigned to placebo, randomization was stratified according to severity on the basis of data obtained during the telephone screening.

### Intervention

Participants received an aqueous extract of house dust mites (*Dermatophagoides pteronyssinus) *in a glycerinated isotonic phosphate buffered solution (Oralgen Mijten, Artu Biologicals, Lelystad, the Netherlands) or placebo treatment consisting of the glycerol solvent. In accordance with the manufacturer's guidelines the treatment period was divided into two phases: a dose escalation phase of 20 days, and a maintenance phase of approximately two years. Treatment started on day one with a single drop. One drop consisting of 0.05 ml corresponds with 35 biological units (BU); the dose was increased by one drop per day until day 20 (20 drops = 1 ml = 700 BU). The maintenance dose was 20 drops (= 700 BU) twice weekly. The drops were administered sublingually and kept there for at least 1 minute before being swallowed. A research assistant instructed the participants and also provided written instructions. Participants, parents, investigators, research assistants and caregivers were blinded to treatment allocation.

### Follow-up

**Figure 1:**
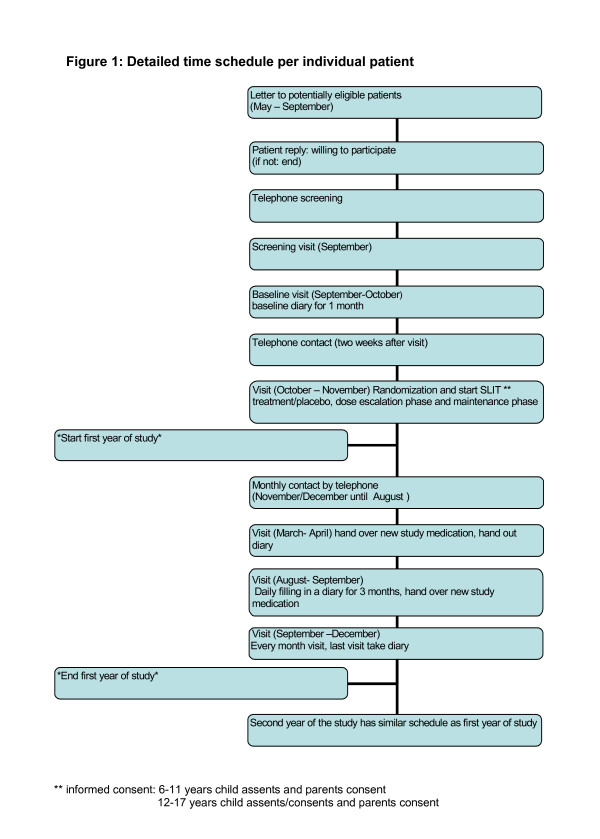
Detailed time schedule per individual patient.

Figure 1 shows the time schedule per individual patient. After randomization children started with treatment for 20 days (dose escalation phase) followed by a maintenance phase of two years. Children filled in a diary during three months (between September and December) after one and two years of treatment (see below). Every month a research assistant completed a questionnaire (conducted by telephone) throughout the entire study period. Over the two years of treatment the total number of planned contacts is 13 home visits and 23 telephone calls.

### Outcome measures

The primary outcome measure for efficacy is the difference between the group receiving SLIT and the group receiving placebo for the total daily mean rhinitis symptom score for four nasal symptoms (see below), assessed through a diary filled in during three months after two years of treatment. In the period of evaluation (September through December), the percentage of days on which the daily symptoms are properly recorded should be at least 50%. For patients who do not meet this criterion in the second year (e.g. dropouts after 1 year) data of the first treatment year will be used. See section Data analysis for further details.

Secondary outcome measures are the difference between the group receiving SLIT and the placebo group for the proportion of symptom-free days, the proportion of rescue medication-free days, use of rescue medication, mean eye symptom score, total symptom score (nasal and eye symptoms), and disease-specific quality of life after two years of treatment. Overall evaluation of the treatment effect will be assessed by patient, parents and research assistant after two years of treatment.

### Assessment of efficacy

Efficacy will be measured by patient-assessed symptom scores. Although nasal, eye, skin and lung-related symptoms have been related to house dust mite allergy, the main allergic symptoms are considered to be the following nasal symptoms: sneezing, itching, watery running nose and blockage. The intensity of these symptoms is subjectively assessed according to a grading scale: 0 = no complaints, 1 = minor complaints, 2 = moderate complaints and 3 = serious complaints; the maximum score is 12. The scores will be assessed daily by the patient and recorded in the patient's diary. The period of measurement will be three months in the period September through December in 2006 and 2007 for the primary outcome measures (first cohort), and in 2007 and 2008 (second cohort); this autumnal period of the year was chosen because it has the highest HDM exposure levels.

### Assessment of safety, tolerability and compliance

Adverse effects will be assessed by patients and parents reporting effects in the diary, or calling the research assistant with complaints, or by the research assistant via a questionnaire filled in during home visits, and by monthly telephone contact. All adverse events reported during the study will be recorded. In case of serious adverse events or persisting allergic symptoms after management according to protocol, the study treatment will be discontinued for these patients. If patients discontinue the study medication, they will be asked to agree to further follow-up according to the study protocol during the remainder of the study period.

Compliance will be measured by self-report of SLIT administration in the diary and by monthly telephone contact, and determined by weighing the returned study medication.

### Sample size calculation

As rhinitis symptoms are the primary outcome measure, this was used for calculating the sample size. A Dutch study on mattress covers provides relevant data for symptom scores in patients with house dust mite allergy (aged 8–50 years). [18] Based on the baseline symptom score in the latter study, and the ability to assess a reduction of at least 30% (proposed by Malling as a clinically relevant reduction) [19], in our study a sample size of 96 patients per group would be required. Taking into account a dropout rate of 25% between randomisation and end of follow-up, this would require 128 patients in each study group. An alternative approach is assuming the nasal score at the last week screening visit to be 4.5 (sd 2.6). A 30% change would provide a delta of approximately 0.5 (generally assumed to be clinically relevant) and require a sample size of 105 per study group (alpha = 0.05 and beta = 95%).

### Quality of life

Rhinoconjunctivitis-specific quality of life will be assessed through the validated Pediatric (6–11 years) and Adolescent (12–17 years) Rhinoconjunctivitis Quality of Life Questionnaire (PRQLQ and AdolRQLQ, respectively) at baseline and after one and two years. [16,17] To establish the presence of lower airway symptoms during the last 12 months at baseline, specific questions on wheezing and dry cough at night were taken from the ISAAC. [15]

### Rescue medication

During the study the use of symptomatic allergy medication is discouraged, especially use of long-acting antihistamines and locally or generally administered corticosteroids. However, rescue treatment is allowed in case of persisting allergic symptoms (levocetirizine tablets, xylomethazoline nasal spray and levocabastine eye drops); the above-mentioned rescue medication will be provided free of charge. In principle, patients are encouraged to use the provided medication only, but are allowed to use their own medication as well. Patients were clearly instructed on the use of rescue medication and other medication, and on how to document entries in the patient diary. For severe or steadily worsening rhinoconjunctivitis symptoms or intermittent asthma the patient should consult his/her physician.

### Data analysis

The treatment effect will be tested at a two-sided significance level of 5%. Statistical comparison between verum and placebo of the mean daily sum score from the diary after two years will be done using Analysis of Covariance. There will be three covariates in this analysis: baseline nasal sum score at entry into study, age of patients, and presence of cat allergy. In case more than one child from the same family has been included in the study and contribute to the final analysis, we will test whether 'family' provides a statistically significant effect (P < 0.20). In that case 'family' will be added as a random effect.

Exploratory subgroup analyses are planned for the difference between placebo and verum regarding the primary outcome according to age and the baseline symptom score (both dichotomized at the median value).

All analyses will be performed according to the intention-to-treat principle, i.e. irrespective of compliance with the prescribed dosing schedule and other treatments, but excluding patients in whom major inclusion criteria were not fulfilled. A per-protocol analysis will include all patients who took at least 80% of the study medication and completed 50% of the diaries.

For this paper, the distribution of age and gender throughout the recruitment period will be compared. All data are presented as summary descriptive statistics: means, standard deviations (SD) or percentages. Statistical analyses were carried out with SPSS version 11.0 and differences of p < 0.05 were considered significant.

## Results

Letters were posted by 226 general practitioners to 5986 children. An answer form was returned by 2555 children; of these, 1072 children responded positively to the letter and 500 of these children were included after the screening by telephone. Finally, 251 children (i.e. only 4.2% of the children selected in general practice) were included in the study.

Table 2 summarizes the main reasons given for not participating in the consecutive recruitment phases. In response to the initial mailing most of those who declined had few or no complaints (48%), or had another allergy (16%). During the telephone screening, those not included had no history of HDM allergy (28%) or a low symptom score (28%). In the last phase of the recruitment (the screening visit) the main reasons for non-participation were no HDM allergy but only grass or tree pollen allergy detected by RAST (33%), and no sensitization to inhalant allergens detectable by RAST (30%).

**Table 2:** Reasons not to participate in the consecutive recruitment phases

Reasons not to participate	Total (n)	Percentage
**Letter returned (n = 1483)**		
Few or no complaints	710	48.4%
Other allergy	240	16.4%
Study too burdensome	202	13.8%
No interest in the study	186	12.6%
No reason	145	9.8%
**Telephone screening (n = 572)**		
No HDM allergy	159	27.8%
Low symptom score (<4/12)	158	27.6%
Not interested in study	57	10.0%
Severe asthma	39	6.8%
Language barrier	27	4.7%
Use of immunotherapy in the last 3 years	19	3.3%
Refusing blood sample to be taken	17	3.0%
Age (out of range)	15	2.6%
Allergic complaints <1 year	12	2.1%
History of severe allergic reaction	9	1.6%
Systemic disease	8	1.4%
Use of nasal corticosteroids 1 month before baseline	7	1.2%
Answer forms received after deadline of inclusion period	45	7.9%
**Screening visit (n = 249)**		
Only grass pollen or tree pollen sensitization	81	32.5%
No sensitization detectable	75	30.1%
Sensitive to pet at home (confirmed by RAST)	60	24.1%
No informed consent	29	11.7%
Use of unallowed co-medication	4	1.6%

Table 3 presents the baseline characteristics of the included patients. The mean age of the participants was 11.8 (SD 3.0) years. A total of 251 children were randomized to treatment or placebo. During the recruitment period nasal complaints were assessed at several time points; this symptom score showed a difference between telephone screening (6.8) and screening visit (4.5). More than half of the children reported wheeze/breathlessness (54%) and dry cough (53%) during the last year. In almost 37% of the children asthma was reported.

**Table 3:** Baseline characteristics of the included children

	Total (n = 251)	Percentage
***Gender***		
Male	149	59.4%
Female	102	40.6%
		
***Age***		
Mean (SD) in years: 11.8 (3.0)		
6–11 years	122	48.6%
12–17 years	129	51.4%
		
***Physical characteristics***		
Weight in kg: mean (SD)	47.5 (15.3)	
Height in cm: mean (SD)	154.6 (17.1)	
		
***Season with most complaints of allergy***		
Spring	35	13.9%
Autumn	14	5.6%
Spring and autumn/entire year	201	80.1%
		
***Nasal symptoms (scale 0–12)***		
Telephone screening: mean (SD)	6.8 (2.1)	
Screening visit in last 3 months: mean (SD)	5.8 (2.3)	
Screening visit in last week: mean (SD)	4.5 (2.6)	
		
***Asthma***		
Asthma present	92	36.7%
Asthma medication	99	39.4%
		
Wheeze/breathless – ever	154	62.3%
Wheeze/breathless – last year	131	53.9%
Dry cough at night – last year	130	52.6%
		
***Sensitization***		
One allergen (monosensitized for HDM)	58	23.1%
Two allergens	67	26.7%
Three allergens	72	28.7%
Four allergens	54	21.5%
		
***Sensitization to both HDM and***		
Grass pollen	179	71.3%
Tree pollen	108	43.0%
Cat dander	85	33.9%

The majority of the children (77%) were multisensitized. Roughly similar proportions of children were diagnosed as being sensitized to one, two, three or four common allergens. Of the included children, 71% was sensitized to both HDM and grass pollen, followed by tree pollen in 43%, and cat dander in 34% of the children.

Table 4 shows the distribution of age and gender during the recruitment process. Of almost 6,000 children, 51% was male and 40% was aged 6–11 years. In the final recruitment phase, 251 children were included in the study. The distribution of age (6–11 years, p = 0.006) and gender (boys 59%, p < 0.025) of the children included in the present study is significantly different from those who initially received the invitation letter.

**Table 4:** Distribution of age and gender during the recruitment phases

	**Total**	**Male**		**Age group** **6–11 years**	
	**n**	**n**	**%**	**n**	**%**
**Total mailed**	5986	3066	51.2%	2369	39.6%
**Letter returned (irrespective of answer)**	2555	1331	52.1%	1036	40.5%
**Letter returned positive response**	1072	592	55.2%	471	43.9%
**Telephone screening positive**	500	279	55.8%	214	42.8%
**Screening visit positive**	251	149	59.4% ^1^	122	48.6%^2^

1: p = 0.006 (compared with 5986 children who were initially contacted)

2: p = 0.025 (compared with 5986 children who were initially contacted)

## Discussion

This is an ongoing randomized double-blind placebo-controlled trial to establish the efficacy of sublingual immunotherapy with house dust mite allergen in children in primary care. Because the effectiveness of SLIT is still under discussion (mainly due to inconclusive quality/methodology of the published trials), the present long-term study is expected to provide useful information about SLIT with house dust mite allergen in primary care.

Although the distribution of age and gender of the participating children is significantly different from those contacted in the first recruitment phase, the difference is relatively small and age and gender groups are adequately represented; therefore, this difference should not affect the generalizability of the results of the trial.

### Strengths and weaknesses

The importance of the methodology and quality of immunotherapy trials has been documented. [19] The present study has a baseline assessment and complies with other recommendations: i.e. placebo-controlled, double-blind, randomized, adequate sample, sufficient duration of treatment, patients selected according to predefined clinical criteria, and clearly defined primary and secondary outcomes.

Most related studies have been performed in a hospital setting, [20,21] so that the results may not be applicable to the general population. Therefore, our study is designed to evaluate – in a primary care setting – the efficacy and safety of SLIT in children and adolescents with house dust mite-induced allergic rhinitis.

The ARIA guidelines propose that SLIT can be administered to young patients if these children are carefully selected with rhinitis, conjunctivitis and/or asthma caused by pollen and mite allergy. [13] By recruiting young children from a primary care setting (according to our methodology) the included children will meet this recommendation.

Most earlier studies failed to report on the phase prior to randomization, whereas the present study reports the reasons given not to participate and possible selection bias.

According to the WAO Task Force, the ideal efficacy study of specific allergen immunotherapy should be performed in monosensitized patients or in patients concomitantly sensitized to noncross-reacting allergens. [22] It is reported that single-allergen-specific immunotherapy may prevent sensitization to other airborne allergens in monosensitized children. [1,3,23] In our study we included both monosensitized and multisentized children; the majority was multisensitized and only 23% was monosensitized. We believe that this will increase the generalizability of the study results to a wider range of patients.

Many clinical trials face recruitment problems and have to approach many patients in order to include only a small proportion. [24,25] In a survey of 78 studies in Dutch primary care, a median of 87% of planned patients was recruited. [26] In the present study 98% of the target sample size was recruited.

## Conclusion

Our study was designed in accordance with recent recommendations for research on establishing the efficacy of sublingual immunotherapy; 98% of the target sample size was reached. This study is expected to provide useful information on the position of SLIT with house dust mite allergen in primary care; results on the efficacy and safety of SLIT should be available by 2010.

## Competing interests

As employees of Erasmus MC, MYB and JCvdW obtained research funding from Artu Biologicals, which included the present study. CMAdB, HM and ER were employed by Erasmus MC through these funds.   JCdJ received project funding from GlaxoSmithKline, Merck, Sharpe & Dohme, Roche and Friso Foods, and lectured at scientific meetings on request of GlaxoSmithKline, Novartis and Merck, Sharpe & Dohme. All payments went to the Erasmus University Medical Center, Pediatric Research Holding. H. de Groot declares to have no conflict of interest with the pharmaceutical world. R. Gerth van Wijk received fees for lectures and expert panel participation from Allmiral, Alcon, Merck Sharp & Dome, Novartis, Stallargènes and UCB. 

## Authors' contributions

CMAdB carried out all analyses and drafted the manuscript. All other authors read and approved the final protocol and the manuscript. JCvdW drafted the protocol and supervised the study.

## Pre-publication history

The pre-publication history for this paper can be accessed here:


